# Supplementary material for: Digestion Profiles of Protein in Edible Pork By-Products
Source: Foods. 2022 Oct 13;11(20):3191. doi: 10.3390/foods11203191 (PMC9602065; doi:10.3390/foods11203191)
Supplement: Supplementary file 1 [file foods-11-03191-s001.zip › foods-1913399-supplementary.pdf]

Table S1. Liberation of potentially bioactive peptides after the *in vitro* gastrointestinal digestion. The bioactive probability which was calculated online (http://distilldeep.ucd.ie/PeptideRanker/), and peptides with the top 5 highest score in the gastrointestinal digest of each sample were illustrated.

**Identified sequences**	**Mass（Da）**	**Origin**	**Bioactive Probability**
Tenderloin, average bioactive probability=0.336			
GVGAGVPGFGVGAGVPGF	1500.773	Elastin	0.881
LGVPFAKPPLGSL	1294.765	Carboxylic ester hydrolase	0.858
ALRGPAGPMGL	1038.564	Collagen type V	0.857
GVGGVGGLGVGGLGAVPGAGAF	1724.921	Elastin	0.852
GVTRPFPLGGVAPRPGFGLS	1981.090	Elastin	0.850
Liver, average bioactive probability=0.386			
FGVGGVGGLGVGGLGAVPGAGA	1724.921	Elastin	0.932
PAGVPGFGVGAGVPGFGVG	1597.825	Elastin	0.925
GVTRPFPLGGVAPRPGFG	1780.974	Elastin	0.873
PGPMGPSGPR	951.460	Collagen preproprotein	0.865
AGVPGFGVGAGVPGF	1287.661	Elastin	0.859
Heart, average bioactive probability=0.262			
GGAPSFPLGSPL	1098.571	Desmin	0.925
LRSPSWDPF	1103.54	Heat shock protein beta-1	0.854
SVPGPMGPSGPR	1137.560	Collagen preproprotein	0.780
TFGGAPSFPLGSPL	1346.687	Desmin	0.775
FPSIVGRPR	1027.593	Actin	0.763
Tripe, average bioactive probability=0.385			
IKAPMFSWPR	1231.654	Carbamoyl-phosphate synthase	0.904
LGVPFAKPPLGSL	1294.765	Carboxylic ester hydrolase	0.858
LGVPFAKPPLGSLR	1450.866	Carboxylic ester hydrolase	0.842
WLPVGPHIMGK	1233.669	Methyltransferase like 7B	0.837
ARSGNPNGEGLPHWPM	1718.795	Carboxylic ester hydrolase	0.828
Skin, average bioactive probability=0.263			
PVDLGKWSGPLSL	1367.745	F1RKG8	0.796
FDKPVSPLL	1014.575	Creatine kinase	0.763
AGNPDLVLPVPAF	1308.708	2-phospho-D-glycerate hydrolyase	0.756
NQKIFDLRGKFKRPTL	1960.137	Troponin	0.751
VVYPWTQRF	1194.619	Hemoglobin	0.712
